# RNA-based characterization of ribosomally active microbial communities during beef slaughtering

**DOI:** 10.3389/fmicb.2026.1946469

**Published:** 2026-09-30

**Authors:** Muhammad Sharjeel Chaughtai, Franz-Ferdinand Roch, Sarah Thalguter, Tea Movsesijan, Beate Conrady, Monika Dzieciol, Martin Wagner, Evelyne Selberherr

**Affiliations:** 1 Centre for Food Science, Clinical Department for Farm Animals and Food System Transformation, University of Veterinary Medicine Vienna, Vienna, Austria; 2 Centre for Veterinary Public Health and One Health, Clinical Department for Farm Animals and Food System Transformation, University of Veterinary Medicine Vienna, Vienna, Austria; 3 Clinical Centre for Population Medicine in Fish, Pig and Poultry, Clinical Department for Farm Animals and Food System Transformation, University of Veterinary Medicine Vienna, Vienna, Austria; 4 Austrian Competence Centre for Feed and Food Quality, Safety and Innovation FFoQSI GmbH, Tulln, Austria; 5 Department of Veterinary and Animal Sciences, University of Copenhagen, Copenhagen, Denmark

**Keywords:** 16S rRNA cDNA sequencing, beef, *Escherichia-Shigella*, *Mycoplasma*, *Pseudomonas*, slaughterhouse

## Abstract

**Inroduction:**

Microbiological contamination on beef carcasses shortens shelf-life and poses substantial food-safety risks. The application of RNA-based analysis nowadays enables insights into the potentially active fraction of microbial communities by targeting ribosomal RNA rather than DNA.

**Methods:**

Here, we profiled the rRNA-derived microbiota present at three critical stages of cattle slaughter, skinning, splitting, and chilling, in an Austrian slaughterhouse, using 16S rRNA cDNA sequencing (Illumina MiSeq).

**Results:**

While microbial alpha diversity was similar across the three processing stations, PERMANOVA revealed a statistically significant effect of the processing step on microbial community composition. Carcasses at skinning and chilling harbored high relative abundances of rRNA-derived *Pseudomonas* and the *Escherichia-Shigella* group. The high relative abundance of *Pseudomonas* spp., a psychrotrophic spoilage-associated genus, suggests potential metabolic activity and a possible contribution to quality deterioration at low temperatures, while the *Escherichia-Shigella* group is commonly used as an indicator of possible fecal contamination and hygiene deficiencies. *Eperythrozoon (Mycoplasma)* was more abundant at the splitting stage and, to a lesser extent, at the chilling stage, suggesting that some cattle may enter the slaughter already colonized and facilitating cross-contamination during carcass manipulation. The subsequent decline during chilling aligns with the cold sensitivity and limited environmental persistence of *Eperythrozoon (Mycoplasma)*.

**Discussion:**

These findings demonstrate the value of RNA-based approaches for delineating the active fraction of carcass-associated microbiota, with potential to inform interventions that enhance meat hygiene and safety.

## 1 Introduction

Beef is a nutrient-dense food that can contribute to dietary intake of protein and selected micronutrients, including iron, zinc, selenium, omega-3 fatty acids, and vitamin B12 (Ruxton and Gordon, 2024). However, beef production and consumption are associated with substantial environmental, health, and ethical challenges. Beef production is resource-intensive, requiring large amounts of land, water, and feed, and it contributes disproportionately to greenhouse gas emissions, deforestation, and biodiversity loss (Godfray et al., 2018). These impacts are compounded by food loss and waste along the supply chain, much of which stems from microbial contamination (e.g., *Pseudomonas* spp., *Clostridium* spp., *Brochothrix thermosphacta*) that drives spoilage, reducing food quality and shelf life, consequently resulting in financial losses and consumer rejection (Ercolini et al., 2006; Pellissery et al., 2020; Tesson et al., 2020; Uhlig et al., 2024). The same contamination can also cause foodborne illness (e.g., *Salmonella* spp. and *Listeria monocytogenes*), with animal-source foods, including beef, representing important sources of outbreaks (Holst et al., 2025). Consequently, improving hygiene and microbial control is central to reducing waste and risk and to evaluating the overall sustainability of beef production alongside its nutritional benefits (Hubbart et al., 2023).

A detailed characterization of the microbial communities associated with beef is essential to guide these efforts. Traditional culture-based methods allow the isolation of pure strains, enable phenotypic characterization, and remain the gold standard for antimicrobial susceptibility testing. However, these approaches are inherently limited, as a substantial proportion of microorganisms, often referred to as the ‘viable but non-culturable’ (VBNC) fraction, cannot be grown under standard laboratory conditions, resulting in an incomplete representation of microbial diversity (Kang et al., 2020). On the other hand, culture-independent techniques, particularly nucleic acid-based sequencing approaches, provide a more comprehensive view of the microbial diversity/communities (Rovira et al., 2023). Microbiological analysis has been transformed by next-generation sequencing (NGS) technologies, by enabling high-throughput and detailed characterisation of microbial populations. The use of conserved regions for primer binding, combined with taxonomically informative hypervariable regions, has made 16S rRNA gene amplicon sequencing one of the most effective methods for assessing bacterial community composition at the genus level (Satam et al., 2023). However, a key limitation of DNA-based methods is their inability to distinguish between living and dead cells, and residual DNA. In contrast, rRNA-based approaches target ribosomal RNA and can provide additional information on the potentially active fraction of microbial communities (Ceuppens et al., 2014; Mann et al., 2015; Wang et al., 2023). Detecting rRNA-derived microbiota could be crucial for food safety assessment, as potential metabolically active bacteria are responsible for spoilage and disease. Characterizing the rRNA-derived microbiota on beef carcasses can yield valuable information for enhancing/improving hygiene practices, identifying contamination sources, and evaluating the effectiveness of decontamination strategies. By reducing microbial transmission and spoiling, such characterization can enhance product quality, support targeted interventions and ultimately contribute to improved public health outcomes (Hultman et al., 2020; Sequino et al., 2022).

## 2 Materials and methods

### 2.1 Sampling, RNA extraction and cDNA synthesis

Samples were collected from bovine carcasses at three processing stages: skinning (immediately post-skinning), carcass splitting (immediately post-splitting), and chilling (from carcasses held in the cooling chamber for >24 h) within a single sampling day at an IFS-Food-certified Austrian slaughterhouse (International Featured Standards)^1^. Chilled carcasses had originated from animals processed on the previous slaughter day, consistent with the >24 h chilling period. Surface scraping samples were obtained from carcasses at skinning (*n* = 9), splitting (*n* = 10), and chilling (*n* = 9) (scraped area: 10 cm^2^, sampling site: upper rump) using a sterile disposable scalpel for each sampling. As the sampling procedure was based on standardized surface area rather than tissue mass, the recovered material was not weighed. Carcasses were selected from different slaughter lots and originated from different farmers. Sampling was conducted early in the first slaughtering shift. Immediately after sampling, each sample was preserved in 1 mL RNAlater® (Invitrogen, USA) to stabilize RNA and stored at −80°C. Prior to nucleic acid extraction, samples were thawed at room temperature, vortexed for 1 min, and centrifuged at 8,000 × g for 5 min to pellet cellular material. Following centrifugation, floating fat and visible tissue fragments remaining on the surface were carefully removed. To improve pellet recovery before proceeding with extraction, the samples were centrifuged again at 8,000 × g for 5 min, followed by removal of the supernatant. RNA was then extracted from the resulting pellet using the TRIzol® reagent protocol (Invitrogen, USA) according to the manufacturer’s instructions. The extracted RNA was eluted in 14 μL of RNase-free water. Residual DNA was removed using the TURBO DNA-free™ Kit (Thermo Fisher Scientific, Vienna, Austria) according to the manufacturer’s instructions. To assess residual DNA contamination, 1 μL of each RNA extract was subjected directly to PCR targeting the V1–V2 region of the 16S rRNA gene using primers F27 (5′-AGA GTT TGA TCC TGG CTC AG-3′) and R357 (5′-CTG CTG CCT YCC GTA-3′). Samples showing amplification (*n* = 14) were subjected to a second DNase treatment. After that, subsequent PCR analysis confirmed the absence of detectable residual DNA in all samples.

RNA concentration and purity were then assessed using a NanoDrop spectrophotometer (Thermo Fisher Scientific, USA), and RNA integrity was evaluated using an Agilent Bioanalyzer (Agilent Technologies, USA). All RNA samples had an RNA integrity number (RIN) ≥ 7 and were therefore used for downstream cDNA synthesis. 11 μL RNA was used for complementary DNA (cDNA) synthesis using the RevertAid H Minus First Strand cDNA Synthesis Kit (Thermo Fisher Scientific, USA).

Positive and negative controls were included in the PCR amplification. In addition, a reagent negative control was subjected to PCR to monitor potential reagent-derived contamination. The positive controls showed the expected amplification, whereas no amplification was detected in any of the negative controls. As no visible PCR products were obtained from the negative controls, these were not included in the sequencing libraries. No mock community was included to independently assess taxonomic assignment performance of the V3–V4 sequencing workflow.

### 2.2 16S rRNA amplicon sequencing analysis

cDNA synthesized/generated from RNA extracted from carcass scrap samples was used as the template for 16S rRNA amplicon sequencing to target rRNA-derived bacterial communities. The hypervariable V3–V4 region of the 16S rRNA (reverse-transcribed to cDNA) was amplified using the universal primers 341F (5′-CCTACG GGN GGC WGC AG-3′) and 805R (5′-GAC TAC HVG GGT ATC TAA TCC-3′). To enable dual indexing and library preparation, each primer was appended with Illumina overhang adapter sequences: 5′-CGT CGG CAG CGT CAG ATG TGT ATA AGA GAC AG-3′ for the forward primer and 5′-GTC TCG TGG GCT CGG AGA TGT GTA TAA GAG ACA G-3′ for the reverse primer. Amplicon libraries were prepared using the Nextera XT DNA Library Preparation Kit (Illumina, USA) following the manufacturer’s protocol/instructions. The resulting libraries were quantified, normalized, pooled, and subjected to sequencing on an Illumina MiSeq platform using a 2 × 300-base pair paired-end read configuration under standard Illumina operating conditions at BaseClear B. V. (Sylviusweg 74, 2,333 BE Leiden, The Netherlands).

### 2.3 Bioinformatics and statistical analysis

The DADA2 plugin (Callahan et al., 2016) in QIIME 2 (vs.2023.9) (Bolyen et al., 2019) was employed for sequence quality control, including denoising and chimera removal. Based on the quality profiles of the raw reads/per-base quality profiles, forward reads were truncated at 270 bp and reverse reads at 210 bp. Taxonomic assignment was conducted using a pre-trained Naive Bayes classifier, with sequences classified against the SILVA database (138.2) (Bokulich et al., 2018; Kaehler et al., 2019; Quast et al., 2012; Robeson et al., 2021). Following DADA2 denoising and chimera removal, sequences classified as chloroplasts, mitochondria, eukaryotes, or unassigned were excluded. No additional abundance- or prevalence-based filtering was applied to the ASV table prior to diversity analyses.

Processed data were exported from QIIME 2 and converted using the qiime export and converted with biom convert, respectively. Subsequently, the data were imported into R (v4.4.1) (R Core Team, 2024) for further statistical testing and visualization. The “phyloseq” package (v1.48.0) (McMurdie and Holmes, 2013) was used to manage and integrate sequencing data in R. Alpha diversities were estimated using Hill-Simpson and Hill-Shannon indices calculated with the “iNEXT” package (v3.0.1) (Hsieh et al., 2016) based on samples rarefied to 99% coverage. Group differences in alpha diversity were assessed using Kruskal-Wallis tests followed by Bonferroni-alpha-corrected Dunn’s post-hoc tests for pairwise comparisons (significance thresholds: *p*-value = 0.05 for Kruskal-Wallis; *p*-value of 0.025 for Dunn’s test).

Samples with more than 1,000 reads were rarefied to 99% sample coverage using 100 iterations implemented in the metagMisc package (v0.5.0) (Mikryukov, 2022). These data were used to generate distance matrices using Bray-Curtis dissimilarities (BC), Jaccard indices (JD), and Jensen-Shannon divergence (JSD) and were subsequently utilized for graphical analysis with t-distributed stochastic neighbor embedding (tSNE) to describe the beta diversity of the samples (Rtsne v0.17) (Krijthe, 2015), using 999 iterations.

Perplexity and initial dimensionality parameters were set following the recommendations by (Oskolkov, 2019) and are detailed in the plot description for each individual plot. The same dataset was used to assess beta-dispersion, followed by one-way ANOVA and Tukey’s Honest Significant Difference (TukeyHSD) tests for pairwise comparisons. Additionally, we conducted a PERMANOVA to assess differences between 16S rRNA cDNAs using the vegan package (v2.6–6.1) (Oksanen, 2022), preceded by tests homogeneity of multivariate dispersion using the betadisper function. Differential abundance analysis was performed using the DESeq2 package (v1.44.0) (Love et al., 2014).

## 3 Results

Across all samples, we obtained 555,914 high-quality reads and resolved 5,540 ASVs, assigned to 33 phyla, 301 families and 687 genera (Supplementary Tables S1–S3). Phylum-level composition by processing station is shown in Figure 1A the most abundant genera are summarized in Figure 1B. At the skinning stage, the community was dominated by Pseudomonadota with a median relative abundance of 59.8% and an interquartile range (IQR) 17.9–76.5%, followed by Bacillota (median 14.0%, IQR 6.9–17.9%), Actinomycetota (median 8.0%, IQR 3.6–20.0%), and Bacteroidota (median 6.0%, IQR 4.5–10.6%). Minor phyla, including Verrucomicrobiota, Fibrobacterota and Desulfobacterota were detected at low relative abundances levels. At the genus level, Pseudomonadaceae/*Pseudomonas* and the *Escherichia–Shigella* group represented the principal Pseudomonadota at skinning (*Pseudomonas*: median 5.1%, IQR 1.9–10.5%, prevalence 89%; *Escherichia–Shigella*: median 16.1%, IQR 5.3–21.1%, prevalence 100%). Within Bacillota, most genera occurred at low abundance (medians <0.5%), with the Oscillospiraceae ‘UCG-005’ reaching a median of 4.9% (IQR 0.2–6.8%), but a prevalence of 78%. Actinomycetota were largely represented by Corynebacteriaceae/*Corynebacterium* (median 3.2%, IQR 1.9–8.5%; prevalence ~100%). Complete phylum-, family-, and genus-level profiles, including per-sample values, are provided in Supplementary Tables S1–S3 and family-level patterns are shown in Supplementary Figure S1.

**Figure 1:**
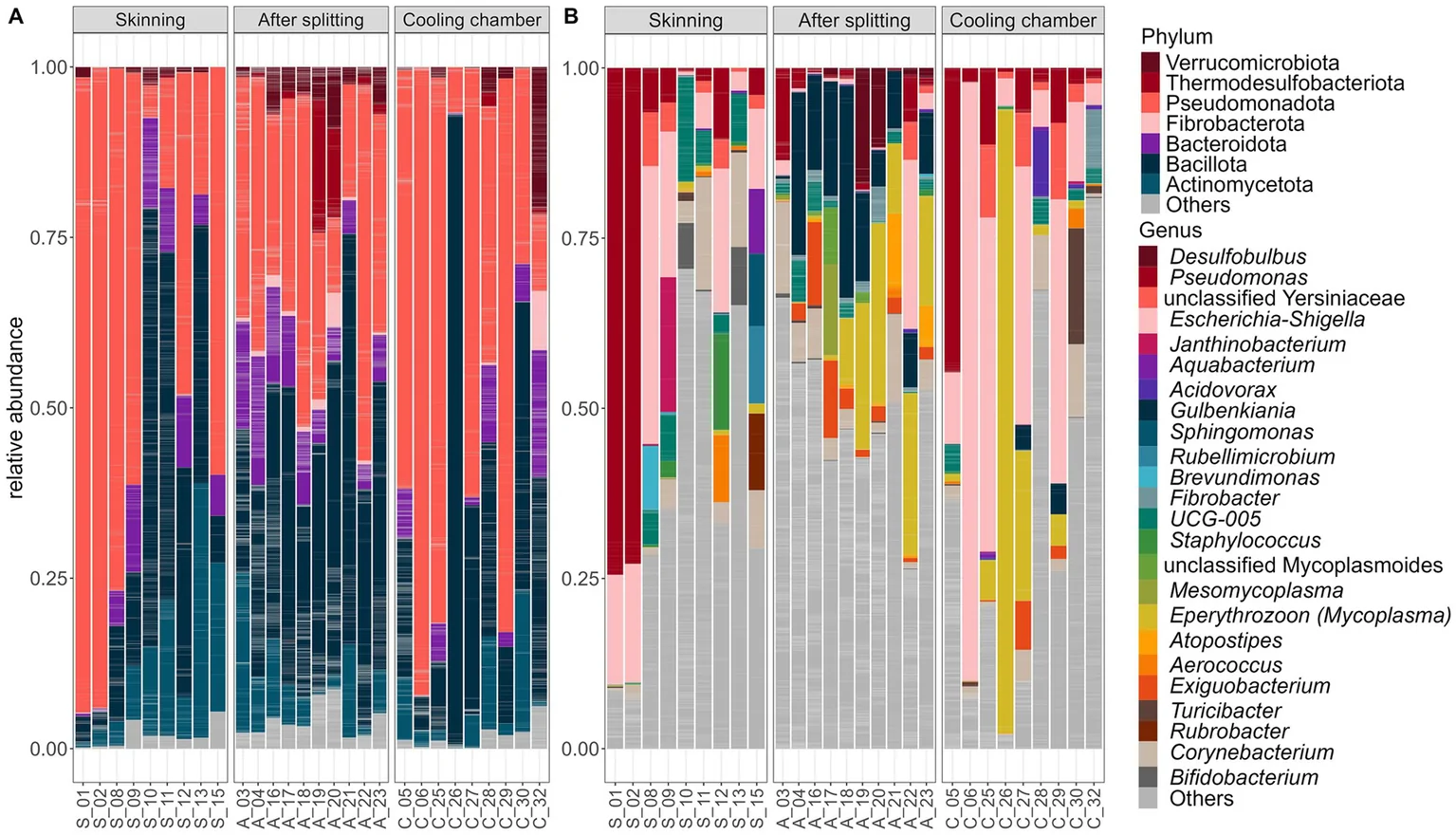
Phylum level **(A)** and genus level **(B)** relative abundance of 16S rRNA-derived ASVs across slaughterhouse processing stages. Relative abundances of bacterial phyla and genera detected on carcass surfaces at different slaughterhouse locations, based on 16S rRNA cDNA sequencing using the Illumina MiSeq platform. Each vertical bar represents individual sample; thin light-grey horizontal lines indicate the boundaries between adjacent ASVs. ASVs assigned to the same phyla or genera share the same color. Phyla/genera reaching ≥ 8% relative abundance in at least one sample are shown individually; all remaining phyla/genera are grouped as ‘Others’.

After splitting, the community composition shifted markedly toward Bacillota which became the dominant phylum (median 36.9%, IQR 29.8–43.3%), followed by Pseudomonadota (median 31.6%, IQR 23.8–38.9%), Bacteroidota (median 8.6%, IQR 4.9–13.3%), and Actinomycetota (median 6.2%, IQR 2.8–10.8%). At the genus level, the most abundant taxa included *Eperythrozoon* (*Mycoplasma*; Bacillota; median 10%, IQR 0.5–20.2%), *Gulbenkiania* (Pseudomonadota; 11.1%, IQR 8.1–16.1%), *Corynebacterium* (Actinomycetota; median 3.8%, IQR 1.8–6.9%), and *Exiguobacterium* (Bacillota; median 2.2%, IQR 1.3–2.9%), all four with a prevalence of 100%, are the most abundant genera. While *Mycoplasma (Eperythrozoon)* and *Corynebacterium* were present in 89 and 100% of the samples after skinning, respectively, *Gulbenkiania* and *Exiguobacterium* could not be detected in any of them.

Carcasses sampled at the chilling stage (>24 h after slaughter) showed a community dominated by Pseudomonadota (median 61.4%, IQR 28.7–80.0%), followed by Bacillota (median 28.5%, IQR 11.2–32.7%), while Bacteroidota were present at lower relative abundance (median 5.5%, IQR 1.1–7.3%). At the genus level, the *Escherichia-Shigella* group was most abundant (median of 11.6%, IQR 5.3–41.7%), followed by *Pseudomonas* (median 2.4%, IQR 2.1–8.1%). All other genera exhibited median relative abundances below 2%.

Alpha diversity was assessed using the Hill-Shannon and Hill-Simpson indices to evaluate microbial community richness and evenness of the rRNA-derived microbial communities across the three processing locations. Boxplots (Figure 2) showed modest variations in diversity among stages, with some groups exhibiting slightly higher median diversity values and narrower interquartile ranges. Within the limits of sample size and observed variability we did not detect statistically significant changes in alpha diversity across processing stations (Kruskal-Walli’s test, *p* > 0.05). Principal coordinates analysis (PCoA) based on BC revealed partial separation of samples by processing location (Figure 3). While substantial overlap was observed between carcasses sampled at the skinning and chilling stations, samples collected from the splitting station tended to occupy a more restricted region of ordination space, indicating greater similarity among these samples compared with earlier and later processing stages. Ordinations generated using Jaccard and Jensen–Shannon distances display broadly similar/comparable patterns (Supplementary Figures S2, S3). Using BC, no significant differences in multivariate dispersion between locations were detected (ANOVA, *F* = 0.51, *p* = 0.607), indicating that differences in community structure were not driven by unequal within-group variability. PERMANOVA identified processing location as a significant factor explaining variation in community-composition (*F* = 2.25, *p* = 0.001), although the effect size was modest (R^2^ = 0.163). Similar results were obtained using Jaccard and Jensen–Shannon distances (Supplementary Table S4).

**Figure 2:**
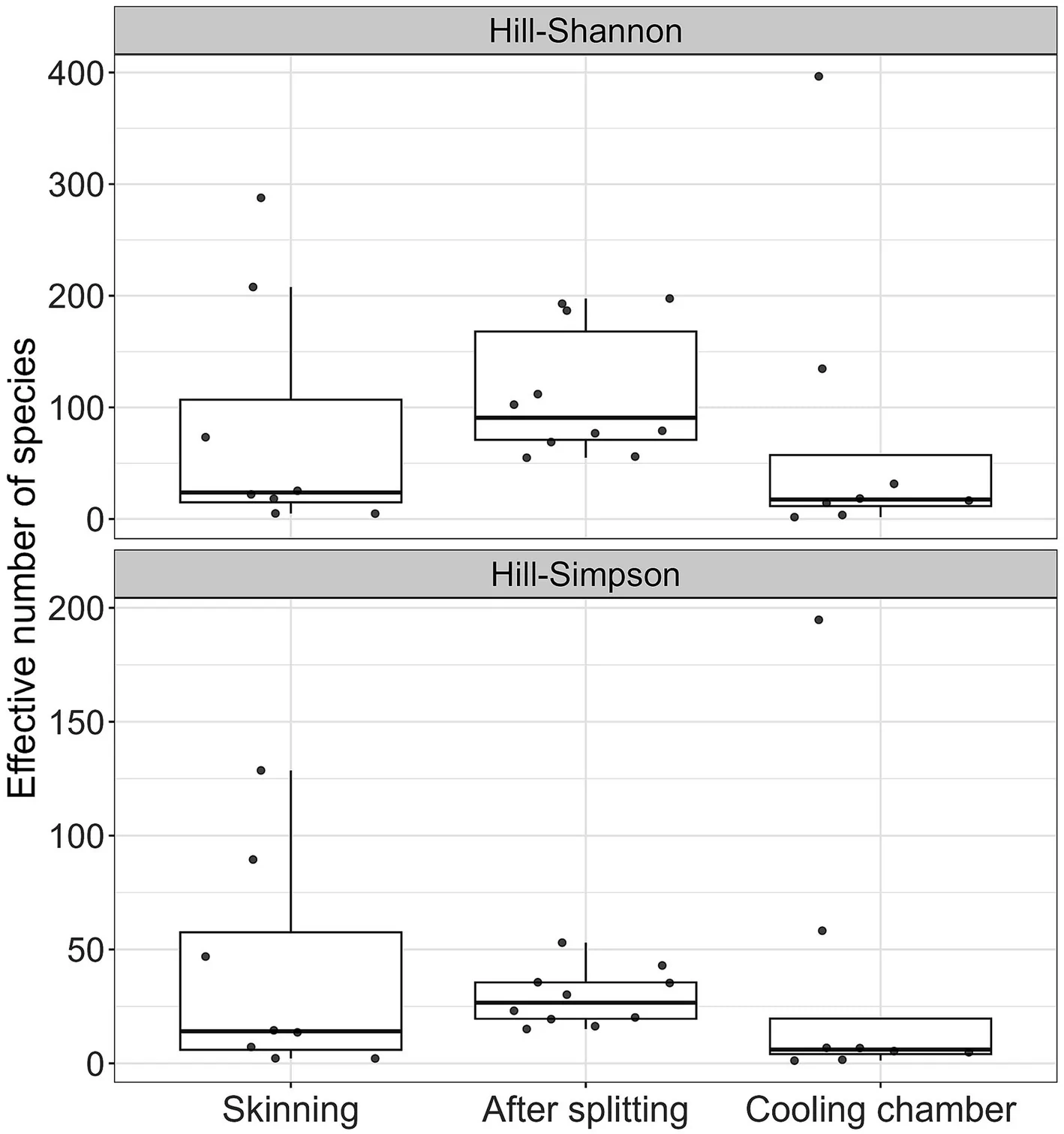
Boxplot comparing Hill-Shannon and Hill-Simpson indices of carcasses sampled from three stations along the slaughter line. Boxes represent the interquartile range (IQR) between 0.25 and 0.75, the horizontal line represents the median and the whiskers ranges to the last data points within the 1.5 times IQR. Overall, the Kruskal-Wallis’s test detected no significant differences between the groups.

**Figure 3:**
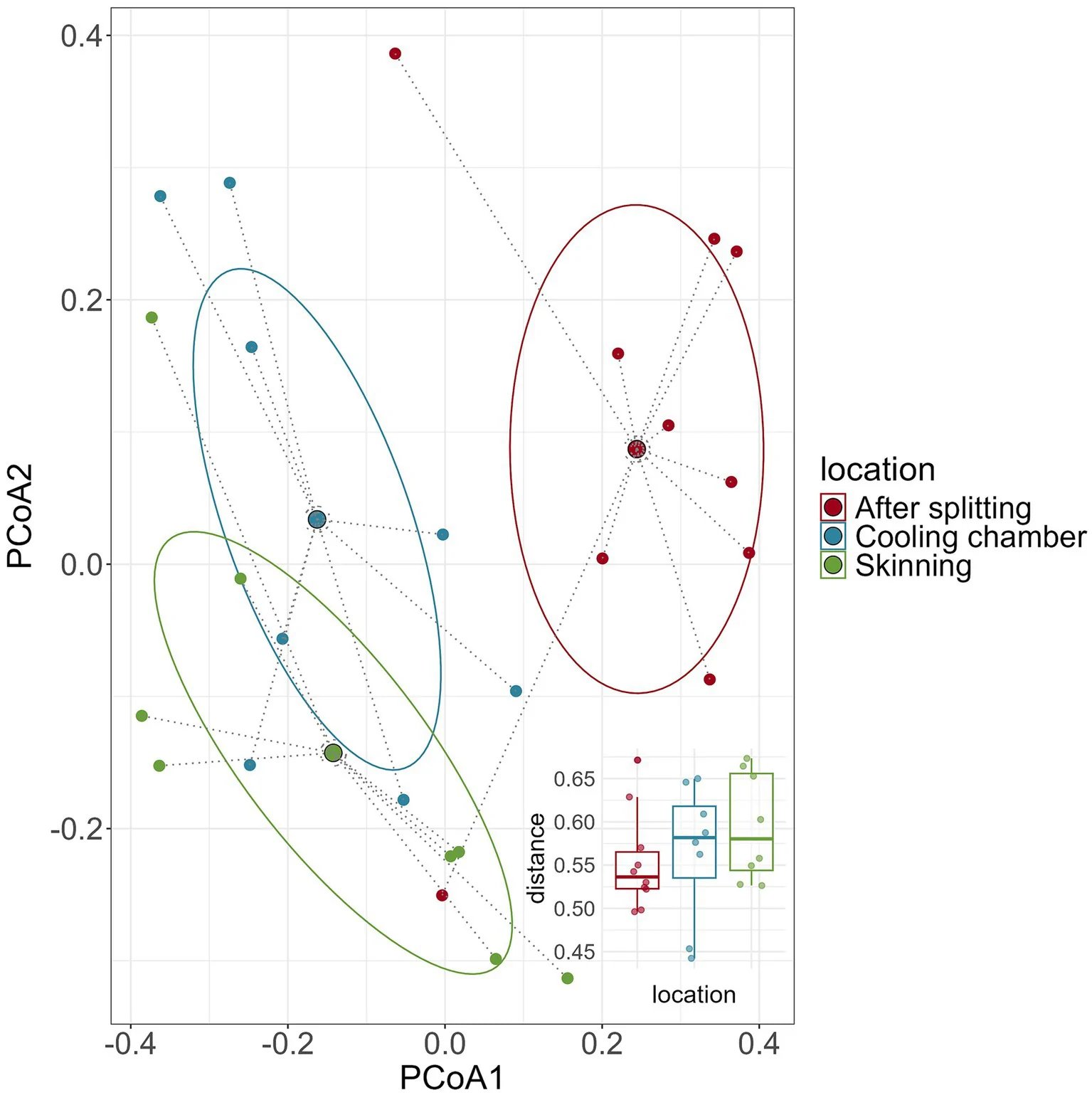
Principal coordinates analysis (PCoA) based on Bray-Curtis distance matrix: this PCoA plot visualizes the multivariate distances among the samples. Each point on the plot represents an individual sample. Larger points with black frames indicate the centroids, representing the mean position within each group. Dashed lines connecting each sample to its respective centroid illustrate the deviation from the centroid, assessing the dispersion within each group. Ellipsoids drawn around the groups provide a spatial interpretation of group dispersion and overlap. The size and shape of these ellipsoids reflect the variance and correlation structure within the groups as captured by the principal coordinates. An inset in the plot visualizes the distribution of distances from individual samples to their centroids, highlighting within-group variability.

To identify taxa whose relative abundance differs significantly between processing stations we used DeSeq2 and calculated log_2_ fold differences in abundance and adjusted *p*-values (Figure 4). Several genera were significantly enriched at the splitting stage, including *Gulbenkiania*, *Exiguobacterium*, *Tepidiphilus*. In contrast, *Halomonas* and *Sphingomonas* were significantly more abundant at the skinning station and declined after splitting.

**Figure 4:**
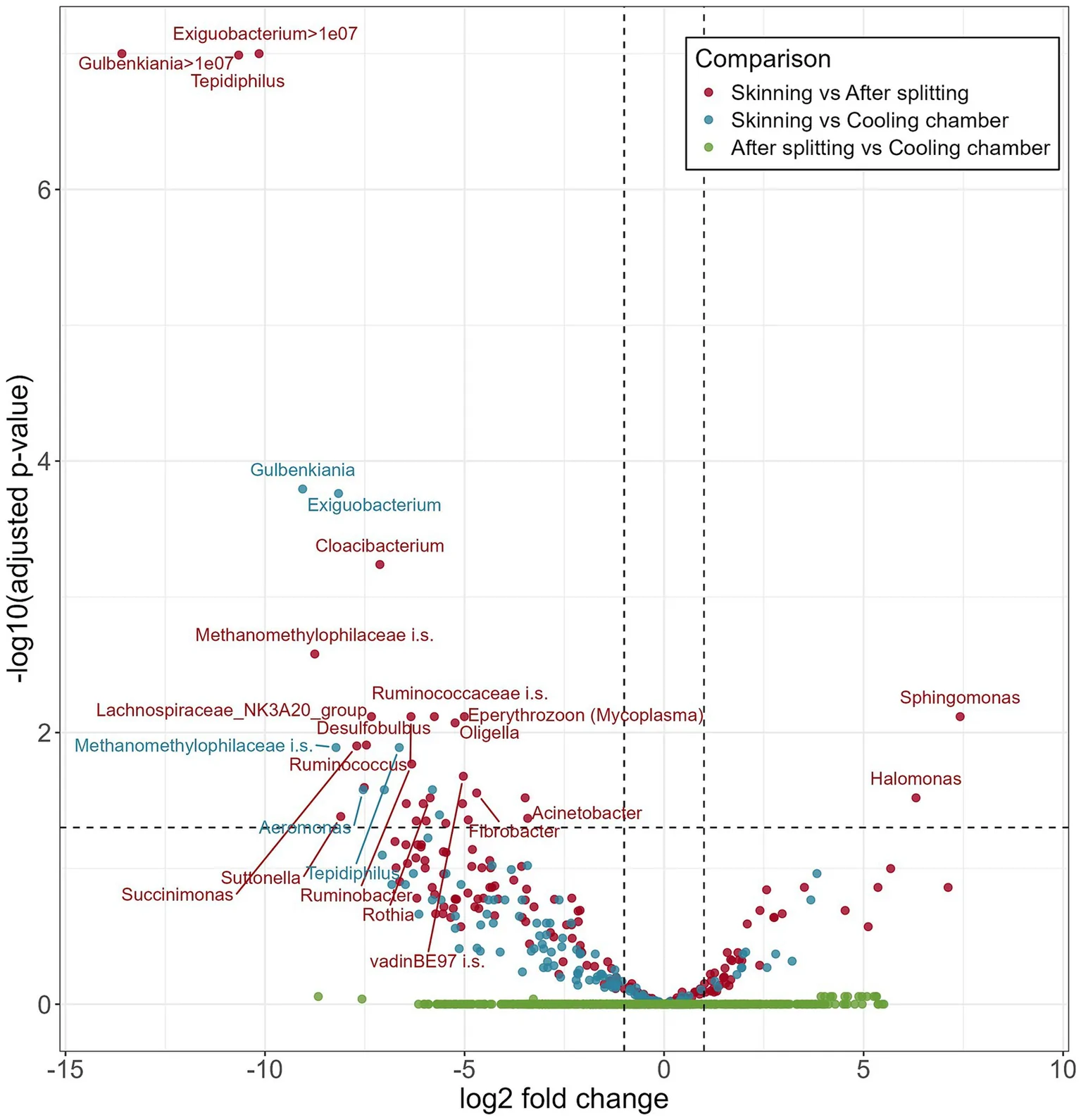
Volcano plots of differential abundance analysis for microbial genera across beef carcass processing stations. In the plot, the *x*-axis (log_2_ fold change) represents both the magnitude and the direction of abundance differences between two stages, while the *y*-axis (−log_10_ adjusted *p*-value) reflects the strength of the statistical evidence after correcting for multiple comparisons. Points higher on the *y*-axis are more statistically significant, with the horizontal dashed line marking the threshold for significance. Vertical dashed lines on the *x*-axis define fold-change cutoffs; taxa plotted beyond these lines represent biologically meaningful abundance differences. Horizontal and vertical dashed lines mark statistical and biological significance thresholds, respectively.

## 4 Discussion

The RNA-based approach employed in the present study targets ribosomal RNA transcripts and therefore reflects the potentially metabolically active fraction of the carcass microbiome, excluding dead cells and extracellular DNA that can contribute to higher apparent richness in DNA-based surveys. An important limitation of the RNA-based approach is that detection of rRNA cannot be considered unequivocal evidence of ongoing metabolic activity. Although RNA is generally less stable than DNA after cell death, ribosomal RNA may persist for some time in recently dead or metabolically inactive cells. Moreover, cellular rRNA abundance is influenced by taxon-specific physiology, growth state, and environmental conditions and therefore does not necessarily correlate directly with metabolic activity. Consequently, the rRNA-derived community characterized here should be interpreted as representing a potentially active or ribosomally active fraction of the carcass-associated microbiota rather than exclusively viable and metabolically active cells. Confirmation of metabolic activity or viability of individual taxa would require complementary approaches, such as cultivation, viability-based assays, or functional RNA analyses.

Previous DNA- and culture-based studies on beef carcasses commonly report genera such as *Pseudomonas*, *Acinetobacter*, *Psychrobacter*, and *Clostridium sensu stricto*, and unclassified Enterobacteriaceae (Botta et al., 2020; Nakamura et al., 2022; Yeom et al., 2024). In contrast, our RNA-community profiling consistently identified *Pseudomonas* and *Escherichia-Shigella* (Proteobacteria) as dominant genera across all processing stations.

Many spoilage bacteria detected later on steaks and other beef products, including *Pseudomonas* spp., and members of the Enterobacterales, are already present early in processing (De Filippis et al., 2013). Across all stages we detected psychrotrophs such as *Pseudomonas* spp., which tolerate cold and can persist even under vacuum or modified atmospheres, signaling both spoilage risk and hygiene gaps (Gram, 1993; Hilgarth et al., 2019; Watson et al., 2021; Yang et al., 2025; Yang et al., 2018). The detection of *Pseudomonas* spp. at the chilling station is critical, as this stage represents a key control point for psychrotrophic spoilage bacteria (Wickramasinghe et al., 2019). Despite rapid temperature reduction intended to limit microbial activity, *Pseudomonas* spp. are well adapted to cold environments and can persist or proliferate, posing a risk of spoilage and reduced shelf life (Chauhan et al., 2023).

According to Regulation (EC) No 2073/2005, Enterobacteriaceae are used as process hygiene indicators for bovine carcasses based on quantitative culture methods. In our study, Enterobacteriaceae were detected on 96.4% of carcasses, with relative abundances exceeding 10% in half of the samples. While these findings indicate a widespread presence of this group, they cannot be directly compared to regulatory thresholds. Nevertheless, given that hides and gastrointestinal contents are major microbial sources in ruminant slaughter (Dong et al., 2020), their occurrence may reflect potential routes of contamination during processing.

Besides common spoilers, we also detected skin related genera, like *Staphylococcus* and *Corynebacterium*, particularly at the skinning stage (88.9 and 100% prevalence, respectively). *Staphylococcus* had a low median relative abundance in the skinning stage (0.4%), but individual samples showed relative abundance up to 14%. In later stages, the median relative abundance was even lower (0.0%). In cattle slaughter hygiene, contamination with *Staphylococcus* is frequently associated with inadequate handling practices, insufficient sanitation of equipment or personnel, and ineffective separation between dirty and clean areas (Diyantoro and Wardhana, 2019). Its absence in the later stages may indicate that hide and handling related contamination was limited beyond skinning. However, as different carcasses were sampled at each stage, inter-animal variability may also have contributed to this pattern. Notably, *E. coli*, which is primarily associated with fecal contamination, remained detectable throughout processing, suggesting that different contamination sources may behave differently across stages. In contrast, *Corynebacterium* had a median relative abundance of 3.2%, which stayed at a relatively high level after splitting (3.8%) but declining in cooling (1.8%). The presence of *Corynebacterium* at all stages can be explained by its ubiquity on bovine hides and in the processing environment. Its continued presence across processing stages may reflect environmental background contamination or inter-carcass variability, rather than persistence on individual carcasses. Even after hide removal, low-level contamination can occur through equipment, air, or personnel contact, allowing this genus to appear consistently across stages.

Genera such as *Halomonas* and *Sphingomonas* were significantly higher after the skinning compared to the other timepoints (Figure 4). We assumed their origin from the animal hide, feces, water, soil, and processing environment, where these genera are frequently encountered. Their decline after splitting may result from carcass washing, trimming, and exposure to equipment surfaces that favor other microbial groups. Further we detected several rumen-related genera such as *Ruminobacter*, *Fibrobacter*, and *Christensenellaceae*. However, their median relative abundances remained consistently low across all processing stages (< 1%). Their low prevalence is consistent with limited transfer from the rumen to the carcass surface and with poor survival of strict rumen anaerobes outside the gut.

After splitting we noted a marked shift of the microbial community compared to the timepoint right after skinning. This is demonstrated in Figures 1B, 4. This was characterized by the abrupt emergence of species such as *Gulbenkiania*, *Exiguobacterium, and Tepidiphilus* at high relative abundances. Since Nakamura *et al*. found in their studies high amounts of *Gulbenkiania* in the sampled carcass washer (Ayaka Nakamura et al., 2023), we assume that a carcass washing or rinsing step, between the skinning and the splitting, might actively introduced these and other genera.

These bacteria are found to be associated with production line and environment, however there are no reports about their spoilage or pathogenic potential. *Desulfobulbus* was absent at the skinning and chilling stations but detected at low abundance during carcass splitting (median 0.6%, IQR 0.1–1.4%). *Desulfobulbus* is an anaerobic sulfate-reducing bacterium commonly found in anoxic aquatic sediments and wastewater biofilms (Jantharadej et al., 2021). Its presence in slaughterhouse wash water and anaerobic digesters from meat processing has been documented (Jabari et al., 2016). Its detection during splitting likely reflects contamination associated with water exposure during processing rather than originating from gut contents, organic residues, or the native skin microbiota. The high relative abundance of sequences taxonomically assigned as *Eperythrozoon* by SILVA 138.2 was notable in several samples (Figure 1B). *A* BLAST analysis of the associated sequence showed 100% nucleotide identity with *Mycoplasma haematobovis*. This hemotropic species adheres to the surface of erythrocytes and typically causes subclinical or mild infections in cattle. Chronic infection with *Mycoplasma haematobovis* can lead to anemia, fever, lymphadenopathy, loss of appetite, and depression (Ferrari et al., 2022). Its detection on carcasses most likely reflects the presence of infected animals at slaughter. The higher detection at splitting (median 10%, IQR 0.5–20.2%) compared with skinning (median 0.1%, IQR 0.1–0.7%) and chilling (median 1.5%, IQR 1.1–5.8%) may therefore be attributed to random distribution of infected animals across sampling time points rather than stage-specific selection. Although *M. haematobovis* is not considered a major concern for food safety, its detection represents an interesting ancillary finding, linking carcass microbiota to the underlying infection status of cattle herds and illustrating how abattoir-based sequencing may serve as a tool for detecting subclinical infections within cattle populations.

As sampling was conducted on a single sampling day but different carcasses were sampled at each processing station, the observed patterns should be interpreted as differences between processing locations rather than as temporal microbial succession within individual carcasses. Importantly, carcasses sampled in the chilling chamber had been processed on the previous slaughter day and had undergone >24 h of chilling, whereas carcasses sampled after skinning and splitting were processed on the day of sampling. Consequently, differences observed at the chilling stage may reflect a combination of chilling conditions, processing-day effects, inter-carcass variability, and facility-associated microbial reservoirs. The study design therefore does not allow these factors to be disentangled. Another aspect is that although the chilling location is characterized by markedly different environmental conditions, particularly lower temperatures, the lack of pronounced community separation suggests that short-term exposure to these conditions may not be sufficient to drive major restructuring of the active microbial community. Instead, processing-related differences are more likely to influence the relative activity and dominance of taxa already present earlier in processing, rather than introducing distinct microbial assemblages. Given that different carcasses were sampled at each station, inter-carcass variability and facility-associated microbial reservoirs likely play a substantial role in shaping the observed beta-diversity patterns.

A further limitation is that, although PCR negative controls and a reagent negative control showed no detectable amplification, negative controls were not sequenced. Therefore, low-level reagent- or laboratory-derived sequence contamination below the detection threshold of the PCR-based control could not be assessed bioinformatically.

Although processing location had a statistically significant effect on microbial community composition, the effect size was modest (PERMANOVA R^2^ = 0.163), indicating that processing stage accounted for only a limited proportion of the overall community variation. This is also consistent with the substantial overlap observed among groups in the ordination analysis. Thus, the observed patterns should not be interpreted as a strong restructuring of the microbial community between processing stages, but rather as stage-associated shifts in the relative composition of the potentially active microbiota.

Overall, active microbial communities reflect a combination of hide-derived, fecal and environmental inputs, with early-introduced taxa persisting through processing despite hygienic barriers. These findings highlight that (i) skinning requires stringent control of hide-derived contamination, (ii) post-splitting stages are vulnerable to water- and equipment-borne introduction, and (iii) chilling must be optimized to limit cold-tolerant spoilage organisms. Targeting these points could provide the most effective leverage for reducing contamination and improving beef carcass hygiene.

## 5 Conclusion

This study demonstrates that RNA-based sequencing provides a clearer view of potentially metabolically active microbes during beef processing. Distinct patterns of key taxa across stations highlight the influence of processing steps and localized environmental conditions. *Pseudomonas* and *Escherichia–Shigella* were consistently detected at the skinning and chilling stations, while showing lower abundance at the splitting stage reflect stage-associated variation rather than temporal changes within individual carcasses being the carcasses different at each processing stage. The incidental detection of *Eperythrozoon* (*Mycoplasma*) sequences further illustrates the broader biological insight this approach can offer. The abrupt appearance of *Gulbenkiania*, *Exiguobacterium*, and *Tepidiphilus* after the splitting stage underscores the dynamic and carcass-dependent nature of microbial inputs during processing. The findings of this study should be interpreted in light of the limitations associated with the relatively small sample size, the single-day sampling design, and the absence of evaluation of environmental factors, including humidity and temperature, that may contribute to microbial shifts during carcass processing. In addition, different carcasses were sampled at each processing station, preventing longitudinal assessment of microbial succession within individual carcasses. Consequently, observed differences may partly reflect inter-carcass variability and facility-specific conditions. Larger, longitudinal, and multi-facility investigations are therefore required to determine whether the activity patterns observed here represent broader features of beef processing microbiomes.

## Data Availability

The datasets presented in this study can be found in online repositories. The names of the repository/repositories and accession number(s) can be found at: https://www.ebi.ac.uk/ena, PRJEB122544.

## Footnotes

1
https://www.ifs-certification.com/en/ifs-portfolio/standards/food-standard

## Supplementary material

SUPPLEMENTARY FIGURE S1 Family level relative abundance of 16S rRNA-derived ASVs across slaughterhouse processing stages. Relative abundance of microbial families isolated from carcasses from different slaughterhouse locations, based on 16S rRNA cDNA sequencing using the Illumina MiSeq platform. Individual ASVs are separated by grey lines. ASVs classified in the same family are filled with the same color. Only families with a minimum of 8% relative abundance in at least one of the individual samples are presented. The rest is grouped in ‘Others’.

SUPPLEMENTARY FIGURE S2 Principal coordinates analysis (PCoA) of carcass samples based on Jaccard distance matrix. This PCoA plot visualizes the multivariate distances among beef samples collected from skinning, after splitting and chilling (cooling chamber) stations. Each point on the plot represents an individual sample. Larger points with black frames indicate the centroids, representing the mean position within each group. Dashed lines connecting each sample to its respective centroid illustrate the deviation from the centroid, assessing the dispersion within each group. Ellipsoids drawn around the groups provide a spatial interpretation of group dispersion and overlap, offering insights into the similarities between different groups. The size and shape of these ellipsoids reflect the variance and correlation structure within the groups as captured by the principal coordinates. An inset in the plot visualizes the distribution of distances from individual samples to their centroids, highlighting within-group variability. No significant differences in beta dispersion between the 16S rRNA transcripts were observed (ANOVA: *F* value 0.125, *p* = 0.883). A PERMANOVA analysis identified the location as a significant factor influencing sample variance (*F* = 1.381, *p* = 0.001) but only explaining 11% of the variance (R² = 0.107).

SUPPLEMENTARY FIGURE S3 Principal coordinates analysis (PCoA) of carcass samples based on Jensen-Shannon divergence distance matrix. This PCoA plot visualizes the multivariate distances among beef samples collected from skinning, after splitting and chilling (cooling chamber) stations. Each point on the plot represents an individual sample. Larger points with black frames indicate the centroids, representing the mean position within each group. Dashed lines connecting each sample to its respective centroid illustrate the deviation from the centroid, assessing the dispersion within each group. Ellipsoids drawn around the groups provide a spatial interpretation of group dispersion and overlap, offering insights into the similarities between different groups. The size and shape of these ellipsoids reflect the variance and correlation structure within the groups as captured by the principal coordinates. An inset in the plot visualizes the distribution of distances from individual samples to their centroids, highlighting within-group variability. No significant differences in beta dispersion between the 16S rRNA transcripts were observed (ANOVA: *F* value 1.198, *p* = 0.320). A PERMANOVA analysis identified the location as a significant factor influencing sample variance (*F* = 3.885, *p* = 0.001) but only explaining 25% of the variance (R² = 0.253).

SUPPLEMENTARY TABLE S1 Sample-wise relative abundance of bacterial phyla identified on beef carcasses at the skinning, post-splitting, and chilling processing stations based on 16S rRNA amplicon sequencing. Median values and interquartile ranges (IQR) of each phyla across processing stages are also presented.

SUPPLEMENTARY TABLE S2 Sample-wise relative abundance of bacterial families identified on beef carcasses at the skinning, post-splitting, and chilling processing stations based on 16S rRNA amplicon sequencing. Median values and interquartile ranges (IQR) for the top 23 families across processing stages are also presented.

SUPPLEMENTARY TABLE S3 Sample-wise relative abundance of bacterial genera identified on beef carcasses at the skinning, post-splitting, and chilling processing stations based on 16S rRNA amplicon sequencing. Median values and interquartile ranges (IQR) for the top 24 genera across processing stages are also presented.

SUPPLEMENTARY TABLE S4 Permutational multivariate analysis of variance (PERMANOVA) and analysis of multivariate homogeneity of group dispersions (betadisper) based on Bray–Curtis, Jaccard, and Jensen–Shannon distance matrices for bacterial communities identified on beef carcasses at the skinning, post-splitting, and chilling processing stations using 16S rRNA amplicon sequencing. Degrees of freedom (Df), R², F-statistics, and *p*-values are presented for each distance metric.
